# Synchronous Adenocarcinoma and Gastrointestinal Stromal Tumor in the Stomach

**DOI:** 10.4103/1319-3767.65196

**Published:** 2010-07

**Authors:** Mohana S. Narasimhamurthy, Gopinathan P. Vallachira, Praveen S. Mahadev

**Affiliations:** Department of Pathology, Sudharma Metropolis, Gastrosurgery, Palakkad District Hospital

**Keywords:** Adenocarcinoma, stromal tumor, synchronus

## Abstract

In recent years, the synchronous occurrence of tumors of different histotypes arising in the same organ has been reported more frequently in the literature. In the stomach, adenocarcinoma has been described with coexisting primary rhabdomyosarcoma, carcinoid, and low-grade B-cell lymphoma of mucosa-associated lymphoid tissue. The simultaneous development of adenocarcinoma and gastric mesenchymal tumor has been documented rarely. We report one such case. A 65-year-old male was diagnosed with a proximal gastric adenocarcinoma and underwent subtotal gastrectomy. Subsequent histopathological examination revealed the presence of another tumor at the gastric antrum. This was a gastrointestinal stromal tumor of low risk category (GIST). The literature has only a few previous reports of this very rare association. It is not known whether this synchronicity is incidental or there is a causative factor inducing the development of tumors of different histotypes in the same organ. Pathologists, oncologists and surgeons should be aware of this interesting condition.

Adenocarcinoma is the most common histological type of gastric tumor. It may coexist with another synchronous tumor of different histological type in a different part of the stomach. Gastric adenocarcinoma may coexist most commonly with lymphoma and less commonly with carcinoid and gastrointestinal stromal tumor (GIST).[1] Rarely, cells of different histological types may intermix and form a collision tumor in the stomach.[2,3] We present here the very rare combination of a synchronous proximal gastric adenocarcinoma and a GIST.

## CASE REPORT

A 65-year-old gentleman presented with a five month history of dyspeptic symptoms, and weight loss. Endoscopy showed ulceration along the lesser curvature of the proximal stomach. Multiple mucosal biopsies were obtained from ulcerated areas and histopathology revealed a poorly differentiated adenocarcinoma. Subsequently, the patient underwent an elective subtotal gastrectomy.

### Pathological findings

**Figure 1:**
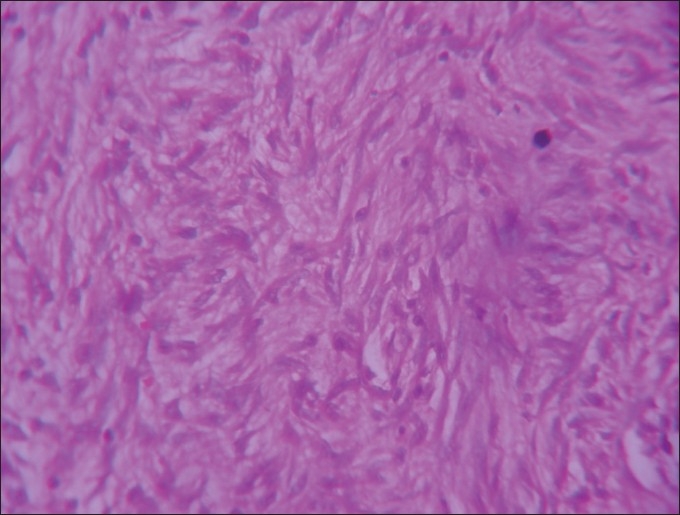
H and E stained, ×40 section shows cytologically bland spindle cells in fascicles

**Figure 2:**
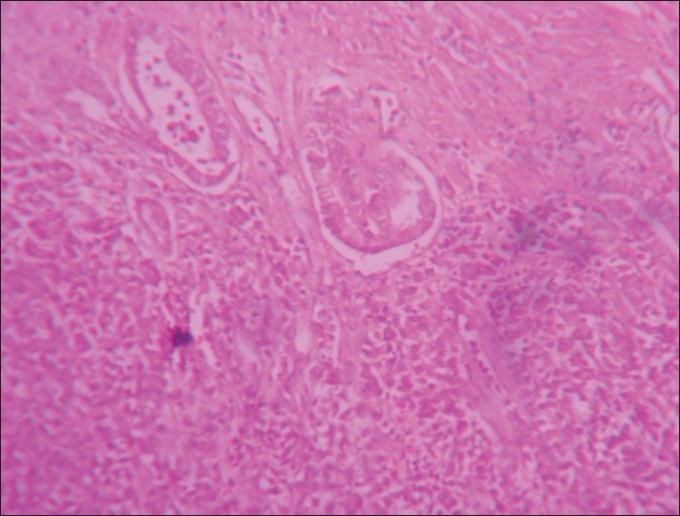
Section from stomach nodule showing glands and sheets of neoplastic cells, H and E, ×40

**Figure 3:**
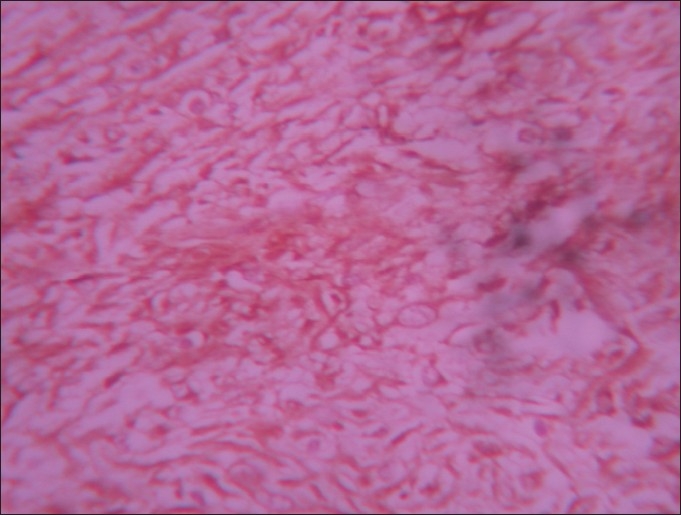
Section from stomach nodule showing diffuse positivity for CD117 of spindle cells, Immunohistochemistry, ×40

Macroscopic examination of subtotal gastrectomy specimen showed firm texture of the proximal stomach and an excavating ulcerative growth measuring 40×30×12mm along the lesser curvature. Interestingly, a nodule of firm white tissue measuring 25 mm diameter, was present adjacent to the ulcerated area. Microscopically, the ulcerated area showed glands and sheets of neoplastic cells which had reached the serosal surface [Figure 1]. Histological examination of adjacent nodule revealed GIST of low risk category [Figure 2], which was composed of cytologically bland spindle cells that were demonstrated by immunohistochemistry to be uniformly positive for CD117 [Figure 3]. Histological assessment of malignancy in GIST is based on tumor size and mitotic count and is classified into four categories as shown in the following Table 1.

**Table 1:** Risk Stratification of GIST

Risk category	Size (cm)	Mitotic count (per 50hpf)
Very low	< 2	<5
Low	2-5	<5
Intermediate	<5	6-10
	5-10	<5
High	>5	>5
	>10	Any
	any	>10

In our case, GIST tumor size was 2.5 cm and mitotic count was less than 5/50hpf. Applying these criteria, GIST was classified into low risk category as mentioned above.

## DISCUSSION

Gastrointestinal stromal tumors (GISTs) are rare mesenchymal neoplasms of the digestive tract. Synchronous occurrence of a gastrointestinal stromal tumor with a tumor of different histogenesis is very rare and has been documented in the literature mainly in case reports. There are only a few previous reports of simultaneous adenocarcinoma and GIST in the stomach.[4–6]

In most of the reported cases of synchronous gastric adenocarcinoma and GIST, the preoperative biopsy fragments showed only adenocarcinoma. GISTs were detected only following laparotomy and examination of the resected specimens. In our case the total gastrectomy was performed for the proximal gastric adenocarcinoma and a small GIST was found incidentally with the histopathological examination of the specimen.

The simultaneous finding of epithelial and stromal gastric tumors raises the question of whether such an occurrence is a simple incidental association or the two lesions are connected by a causal relationship. The suggestion that the stomach harboring a leiomyosarcoma may have a tendency to develop malignant epithelial lesions was put forward by Tada *et al*.[5] Various hypotheses have been proposed regarding the simultaneous development of GIST and adenocarcinoma. Coincidence alone could easily account for such an association, particularly in countries that exhibit high incidence rates of gastric cancer, such as Japan. The possibility that gene mutations might underlie tumor predisposition in patients harboring a double gastric neoplasia cannot be theoretically discarded. Evidence of familial disease was derived in only one case.[5] At present, however, no data are available to support such a hypothesis.

An interesting hypothesis is that a single carcinogenic agent might interact with two neighboring tissues, inducing the development of tumors of different histotypes in the same organ. Experimental evidence for this possibility has been provided. *N*-methyl- *N*’-nitro-*N*-nitrosoguanidine induces the development of gastric adenocarcinomas after oral administration in rats.[5,7] However, when the same compound is combined with agents that alter the gastric mucosal barrier, such as aspirin or stress, leiomyosarcomas develop in conjunction with epithelial tumors.[8] Equally compelling, although also experimental, are reports on the induction of gastric tumors in rats after 9,10-dimethyl-1,2-benzanthracene (DMBA) injection. Whereas administration of DMBA alone induces the development of adenocarcinomas, treatment with DMBA and cellophane plate causes mainly the induction of gastric sarcomas.[9]
